# Host Genome–Metagenome Analyses Using Combinatorial Network Methods Reveal Key Metagenomic and Host Genetic Features for Methane Emission and Feed Efficiency in Cattle

**DOI:** 10.3389/fgene.2022.795717

**Published:** 2022-02-23

**Authors:** Stefano Cardinale, Haja N. Kadarmideen

**Affiliations:** Synomics Ltd, Hanborough Business Park, Long Hanborough, United Kingdom

**Keywords:** methane emission, cattle, combinatorial analyses, synomics insight, rumen microbiome, heritability

## Abstract

Cattle production is one of the key contributors to global warming due to methane emission, which is a by-product of converting feed stuff into milk and meat for human consumption. Rumen hosts numerous microbial communities that are involved in the digestive process, leading to notable amounts of methane emission. The key factors underlying differences in methane emission between individual animals are due to, among other factors, both specific enrichments of certain microbial communities and host genetic factors that influence the microbial abundances. The detection of such factors involves various biostatistical and bioinformatics methods. In this study, our main objective was to reanalyze a publicly available data set using our proprietary *Synomics Insights platform* that is based on novel combinatorial network and machine learning methods to detect key metagenomic and host genetic features for methane emission and residual feed intake (RFI) in dairy cattle. The other objective was to compare the results with publicly available standard tools, such as those found in the microbiome bioinformatics platform QIIME2 and classic GWAS analysis. The data set used was publicly available and comprised 1,016 dairy cows with 16S short read sequencing data from two dairy cow breeds: Holstein and Nordic Reds. Host genomic data consisted of both 50 k and 150 k SNP arrays. Although several traits were analyzed by the original authors, here, we considered only methane emission as key phenotype for associating microbial communities and host genetic factors. The Synomics Insights platform is based on combinatorial methods that can identify taxa that are differentially abundant between animals showing high or low methane emission or RFI. Focusing exclusively on enriched taxa, for methane emission, the study identified 26 order-level taxa that combinatorial networks reported as significantly enriched either in high or low emitters. Additionally, a Z-test on proportions found 21/26 (81%) of these taxa were differentially enriched between high and low emitters (*p* value <.05). In particular, the phylum of Proteobacteria and the order Desulfovibrionales were found enriched in high emitters while the order Veillonellales was found to be more abundant in low emitters as previously reported for cattle (Wallace et al., 2015). In comparison, using the publicly available tool ANCOM only the order Methanosarcinales could be identified as differentially abundant between the two groups. We also investigated a link between host genome and rumen microbiome by applying our Synomics Insights platform and comparing it with an industry standard GWAS method. This resulted in the identification of genetic determinants in cows that are associated with changes in heritable components of the rumen microbiome. Only four key SNPs were found by both our platform and GWAS, whereas the Synomics Insights platform identified 1,290 significant SNPs that were not found by GWAS. Gene Ontology (GO) analysis found transcription factor as the dominant biological function. We estimated heritability of a core 73 taxa from the original set of 150 core order-level taxonomies and showed that some species are medium to highly heritable (0.25–0.62), paving the way for selective breeding of animals with desirable core microbiome characteristics. We identified a set of 113 key SNPs associated with >90% of these core heritable taxonomies. Finally, we have characterized a small set (<10) of SNPs strongly associated with key heritable bacterial orders with known role in methanogenesis, such as Desulfobacterales and Methanobacteriales.

## Introduction

The microbiome has a strong impact in sustainable animal production in the context of feed efficiency, animal health (e.g., antibiotic resistance), and greenhouse gas (GHG) emissions (methane and CO_2_); for a comprehensive overview, see (Beauchemin et al., 2020). Reducing methane emissions from anthropogenic-related sources has been identified as a key area for mitigating climate change with short-to-medium term effects. Nevertheless, methane emissions from livestock are predicted to markedly increase due to an expected doubling in the global milk and meat demand by 2050 (FAO, 2006). Reducing methane emissions may also be tied to improved feed efficiency as methane emission constitutes a loss of dietary energy for the cow. On the other hand, methane production could help the body absorb calories and nutrients by improving fermentation through consumption of excess hydrogen and formate and an increase in acetate production (Hansen et al., 2011). Indeed, genetic correlations of 0.49–0.54 between methane production and milk yield might indicate that genetically selecting for low methane emissions may decrease productivity in dairy cows (Breider et al., 2019). Although the link between microbiota and methane emission is well known, recent studies reveal that host genetics also influence methane emission (Fan et al., 2021) and feed efficiency (Li et al., 2019). The rumen microbiota is surprisingly resistant to changes in substrate (feed) (Montes et al., 2013), rumen transplantation (transfaunation), or treatments introduced as mitigation strategies for methane production, suggesting the existence of a host influence on rumen microbial composition (Weimer et al., 2010). Based on this evidence, genetic selection for low-methane-emitting cows is promising as it is sustainable, persistent, and cumulative over subsequent generations. Despite this, incorporating methane production in a genetic selection program remains challenging partially because the interaction between rumen microbiota and host genetics and physiology remains poorly understood and also because measuring methane production in a manner that reflects the long-term methane phenotype of the animal is difficult (Lovendahll et al., 2018).

Microbiome data are high dimensional and zero inflated (with an excess of zero counts). Furthermore, they are subject to strong total count constrains due to large variability in DNA library sizes and normalization as well as constraints for the maximum number of sequence reads from the sequencing instrument (Rodriguez-R and Konstantinidis, 2014). Although multiple normalization techniques have been developed, none of them really capture the property of scale invariance, known from the concept of compositional data and observations carrying relative information (Weiss et al., 2017). The large number of machine learning (ML) methods applied to the analysis of microbiome data can be largely classified in supervised and unsupervised learning methods. Logistic regression is a statistical method widely used in data science, and it has also been applied to identify microbial signatures that are good predictors of a disease. In one example, Fukui and coworkers used a LASSO logistic regression-based approach to extract a featured group of bacteria for identifying irritable bowel disease (IBD) patients (Fukui et al., 2020). The linear discriminant analysis (LDA) effect size (LEfSe) method proposed by the Huttenhower Lab was developed specifically for biomarker discovery in metagenomic data. This technique performs high-dimensional class comparisons identifying the features that most likely explain the differences between sample groups (Segata et al., 2011). More recently, artificial neural networks (ANNs) and deep learning (DL) have been applied as classification strategies on microbiome data. For example, Lo and Marculescu use an ANN approach on real data sets from the Human Microbiome Project (HMP) (Turnbaugh et al., 2007) and show that it outperforms other methods previously used to classify diseases (Lo and Marculescu, 2019). Ensemble methods, which combine multiple classifiers with the aim of achieving higher classification accuracy, are also successfully applied to microbiome data. Random forest (RF) classifiers, multiple decision trees, and gradient boosting (GB) are only some of the methods reported in dozens of studies currently available on this topic; for a comprehensive review, see Marcos-Zambrano et al., 2021). In one case, GB has been applied to analyze combinations of 16S rRNA, host transcriptome, epigenome, genotype, and dietary data from colonic biopsies of IBD patients and healthy controls showing that, when microbiota information was combined with diet and host genotype information, disease classification improved significantly (Ryan et al., 2020). Among unsupervised learning methods, which try to find apparent patterns in the data without the use of predefined labels, mostly clustering algorithms have been adopted as a preferred strategy for analyzing this type of data. In particular, biclustering, a technique that is widely used for the analysis of gene expression data, is well suited for studies of host–microbiome interactions as it allows us to detect overlapping clusters on both microbes and hosts. This strategy has recently been successfully applied to reveal clusters of bacteria associated with IBD and known gut enterotypes (Zhou et al., 2021).

Whether or not the host influences the rumen microbial community and, consequently, methane production needs to be better elucidated. If reduced methane production is a consequence of poor symbiosis of the host with rumen microbes and, thus, fiber digestibility, there is a risk that genetic selection for hosts with reduced methane production will act against the very symbiosis that has laid ruminants and rumen microbes’ coexistence. Evidence of this has probably been observed in sheep because low-methane animals showed lower feed digestibility than high-methane-emitting animals (Pinares-Patino et al., 2011). In a scenario in which host genetics impose a strong influence on rumen microbial composition, traits influenced by rumen microbes could be improved by using rumen microbial composition as an indicator for genetic selection. However, should host genetics be decoupled from rumen microbial composition, there is a risk of unfavorable side effects from unexpected changes in rumen microbial composition in genetically selected hosts. Here, we hypothesize that 1) the relative composition of the microbiome in the rumen is heritable (i.e., controlled by host genome), and 2) variations in methane emission and feed efficiency are influenced by both the bovine genome and rumen microbial content. To find the determinants orchestrating the impact of changes in the rumen microbial composition on methane production and food efficiency as well the host genetic determinants influencing the changes of the heritable subset of the rumen microbial composition, we used an in-house-developed ML technology based on testing millions of combinations of input predictors through a computationally optimized algorithm that we name Synomics Insights.

### Materials and Methods

#### Study Design

**FIGURE 1:**
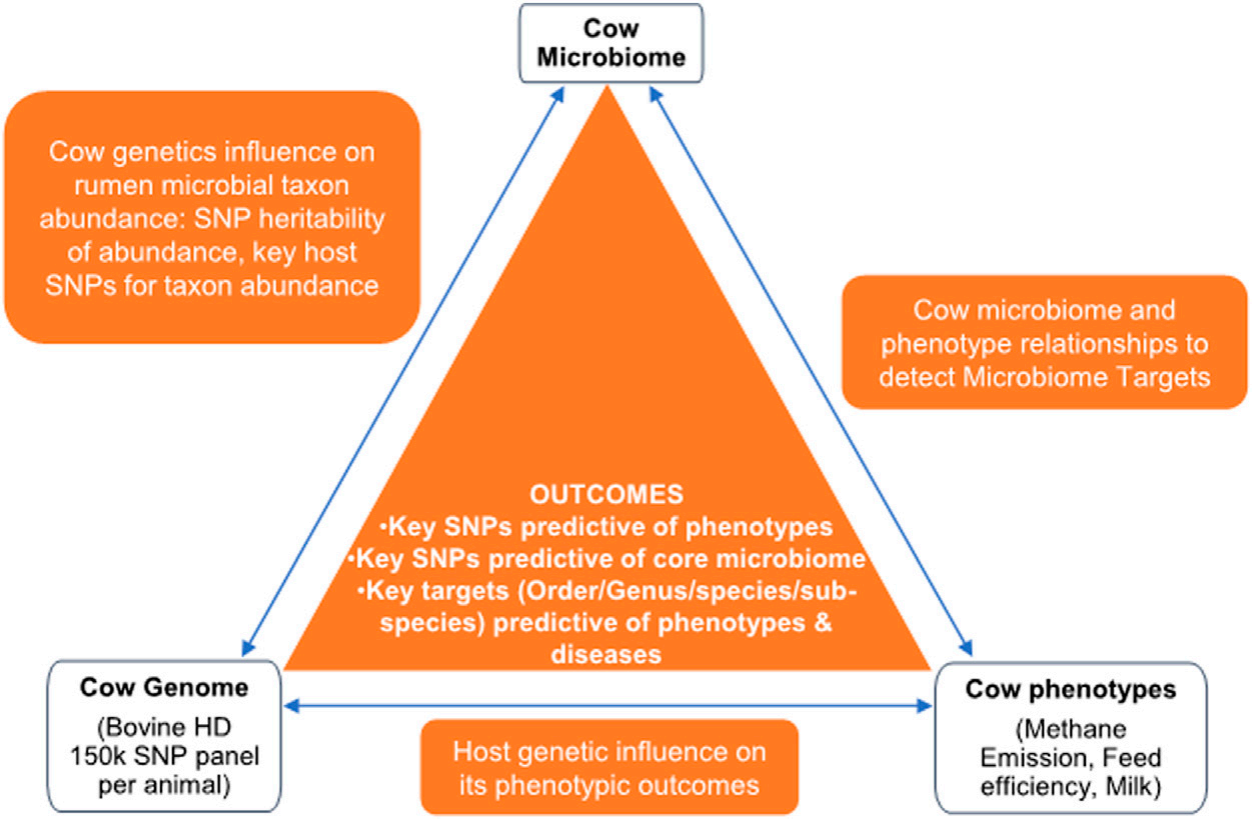
Diagram of Microbiome Systems Genomics concept.

Following the systems genomics concept proposed by Kadarmideen (2014) and the integrative metagenomics approaches described in Suravajhala, Kadarmideen and co-authors (Suravajhala et al., 2016), we developed a study design that integrates host genetics (SNPs), host phenotypes (e.g., RFI, methane emission, milk yield), and metagenome profiles to identify 1) key microbial taxa that influence each phenotype and 2) key genetic factors that determine/influence heritable microbial taxon abundance in rumen (Figure 1). For this study, we utilized a data set from a recent study from Wallace and co-workers (John Wallace et al., 2019). Briefly, 1,016 dairy cows from four European countries have been sampled by amplicon NGS sequencing (Illumina Inc.) of the variable 16S rRNA region and genotyped using the Bovine GGP HD (GeneSeek Genomic Profilers) chip v1 (80K, 200 cows) or chip v2 (150K, 800 cows). 16S rRNA and other microbial marker gene sequences were downloaded from the Short Reads Archive (SRA) under project accession PRJNA517480. We employed MiniKraken DB_8 GB from the publicly available Kraken tool (https://ccb.jhu.edu/software/kraken/). This tool assigns a kmer to the low common ancestor (LCA) of all the genomes containing it. The collective assignment of all kmers then informs the classification of the sequence to the LCA.

#### Preparation of Data sets for Synomics Insights

First, raw Illumina reads were processed to obtain count frequency tables at any given taxonomic level using the platform QIIME2 (Estaki et al., 2020). Our Synomics Insights platform processes ordinal or nominal input data as predictors of a binary response variable. Therefore, count frequency tables were binned, and phenotype continuous data were binarized to make them amenable for processing through our Synomics Insights platform. For binning, we utilized the cut () function in the programming language R, subdividing the values in 10 bins. Binarization of the continuous trait values is described in the main text and employed a combination of linear regression and quantile-based segmentation, which was also performed in R.

Raw genotype data were processed similarly for GWAS and Synomics Insights. Briefly, animals with outlier genetic backgrounds were removed by calculating the first five principal component analysis (PCA) components and identifying outliers. Variants were filtered for minor allele frequency threshold (maf = 0.05), genotype frequency (geno = 0.05), and Hardy–Weinberg disequilibrium (hwe = 0.001). Linkage disequilibrium thinning was done by applying a rolling window and calculating the correlation *r* ^2^ value (--ld-window-r2 0.8). GWAS was performed with PLINK1.9.

#### Heritability

In the estimation of heritability (h^2^) analysis, each microbe is a quantitative “trait” that is encoded by host genetics. For calculating taxon heritability, the taxonomic level was at the order level. The taxa abundance was considered as a quantitative trait and given as individual file input in the Synomics Insights platform. They were first subject to quality control (QC) before heritability analyses as follows. It consists of selecting only those taxa whose frequency is not null in at least 70% or 90% of the 995 animals making up the full set and, in any case, not less than 600 animals. All taxa that do not satisfy this requirement are discarded. Heritability is computed using the software program GCTA (Genome-wide Complex Trait Analysis) (Yang et al., 2011). GCTA is designed to estimate the proportion of phenotypic variance explained by all genome-wide SNPs for complex traits to obtain the SNP-based heritability. First the genetic relationship matric (GRM) is computed between pairs of individuals (flags–grm and -pca 5), that represents the host genome. Then, it is used in the mixed linear model consisting of fixed effects (breed, farm, country, age, parity number) and individual animal genetic effects as random effect to estimate heritability of microbial taxon abundances.

The GCTA method estimates the proportion of additive genetic variance for a taxon trait and, thus, narrow-sense heritability and so should be lower than the *h* ^*2*^ estimate of clonal repeatability. The procedure was required also to set a threshold on heritability (h^2^ ≥ 0.15) for any taxon to be further analyzed using GWAS for determining host genetic influence on taxon abundance.

#### Synomics Insights

The core technology behind Synomics Insights is based on combinatorial analysis aimed at identifying combinations of features that are characteristics of disease vs. healthy individuals or phenotype categories (e.g., occurring in many cases and relatively few controls or found in high vs. low producing animals). These features may include genetic variants (SNPs, CNVs) or other multi-omics features, such as transcripts; CpG sites; or even epidemiological, environmental, or other nongenetic/nonbiological factors. A foundational component of the platform is the same as that used in Precision Life for human biomedical applications (Gardner, 2021). The algorithm is based on constructing combinations of features progressively from low to higher order (so called “layers”) and validating these networks of features using multiple testing correction to adjust the *p*-value of the statistical test including Fisher’s exact test and the Z-score test. For evaluating the predictive ability of classifying cases vs. controls of Synomics Insights features, we used the Youden’s J statistic: ***J = sensitivity + specificity -1***.

### Results

#### Input Data Preparation

All initial 56 phenotypic variables were used as predictors in a linear Lasso regression model with methane, RFI, and milk as independent variables. The LASSO regression shrunk the original set of 56 phenotypes down to 4–10 variables with a nonzero coefficient, and these were further used in a linear regression model to identify the best predictors. Overall, Lasso identified *country* of origin, farm *site code*, and intake phenotypes as common significant predictors of the three traits with the addition of lactation number for milk yield. We used this smaller subset of covariates in a linear regression model to identify which ones are strong predictors of each target treat. We found *country, farm, starch intake, C protein intake*, and *NDF intake* to be the best predictors for milk production (*p* < 10^–16^); *farm, starch intake, dry matter*, and *NDF intake* to be the best predictors of methane emission (*p* < 10^–16^); and *farm, dry matter intake* and *C protein intake* to be the best predictors of RFI (*p* < 10^–16^).

Our Synomics Insights platform can currently only use a binary classification of samples in cases and controls (0/1). Therefore, for each linear regression, we computed the residuals of the trait values using the model predictions. The residuals were then used to binarize the data set and obtain a set of cases and controls. Specifically, we tested the use of percentiles and Z-normalization and found that the two methodologies provided similar outcome with respect to the ability of our Synomics Insights to identify good classifiers of cases and controls among bacterial taxa (Supplementary Figures S1, 2). Given that the use of Z-scores gave larger sample sizes with equally good classification accuracy, we opted to use Z-normalized value and a cutoff ±0.5 sd from the mean for downstream analysis. The final data set comprised the following number of animals roughly equally distributed between cases and controls: 623 animals for methane emission analysis, 604 animals for milk production, and 606 animals for RFI (Supplementary Figure S3).

#### Synomics Insight Networks

**FIGURE 2:**
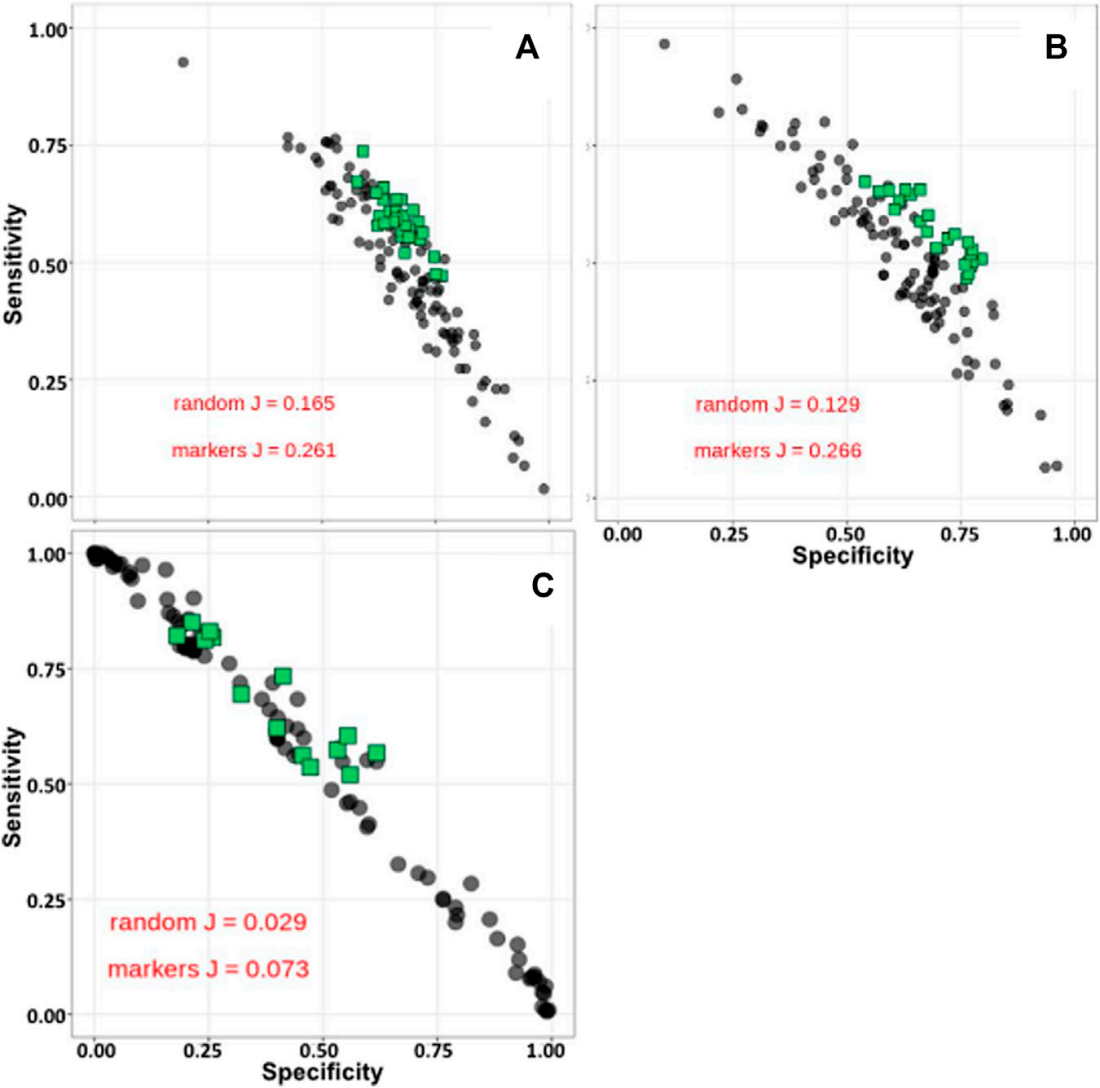
Sensitivity-specificity plots of top taxa from Synomics Insights layer 4 for methane **(A)**, milk **(B)** and RFI **(C)** at Order taxonomic level. J: Youden’s J statistic.

Order and species relative taxonomy abundance levels were used as input to our Synomics Insights platform after relative proportions were binned (see Materials and Methods). It was possible to observe already in the input data a higher abundance of Desulfovibrionales in high compared with low methane emitters and an opposite trend for other orders, including Veillonellales (Supplementary Figure S4). Our engine with this kind of input produces a list of taxon:bin combinations (networks) that are significantly enriched in cases versus controls (see Materials and Methods). The number of combinations depends on the layer from the output result: layer 1 has only one taxon:bin combination, layer 2 has two combinations and so forth; we used outputs up to four layers (Supplementary Figure S5). The size of taxon:bin networks at the order taxonomic level were comparable in size among the three traits: 1,292 for methane, 595 for milk and 1,662 for RFI. Species taxon:bin output at higher network level (layer 4) varied more as RFI did not give any output, whereas methane gave 21 and milk 12 combinations. The top taxon:bin combinations called by Synomics Insights at any given layer were significantly better predictors of cases and controls compared to random (tested by bootstrap, metric was the Youden’s J statistic) for methane and milk (Figures 2A,B respectively). RFI analysis also found some, but not all, top taxon:bin combinations that were good predictors of RFI (Figure 2C). Species-level taxon:bin combinations from our Synomics Insight platform were also very good predictors of methane and milk output (Supplementary Figure S6) compared with random.

**FIGURE 3:**
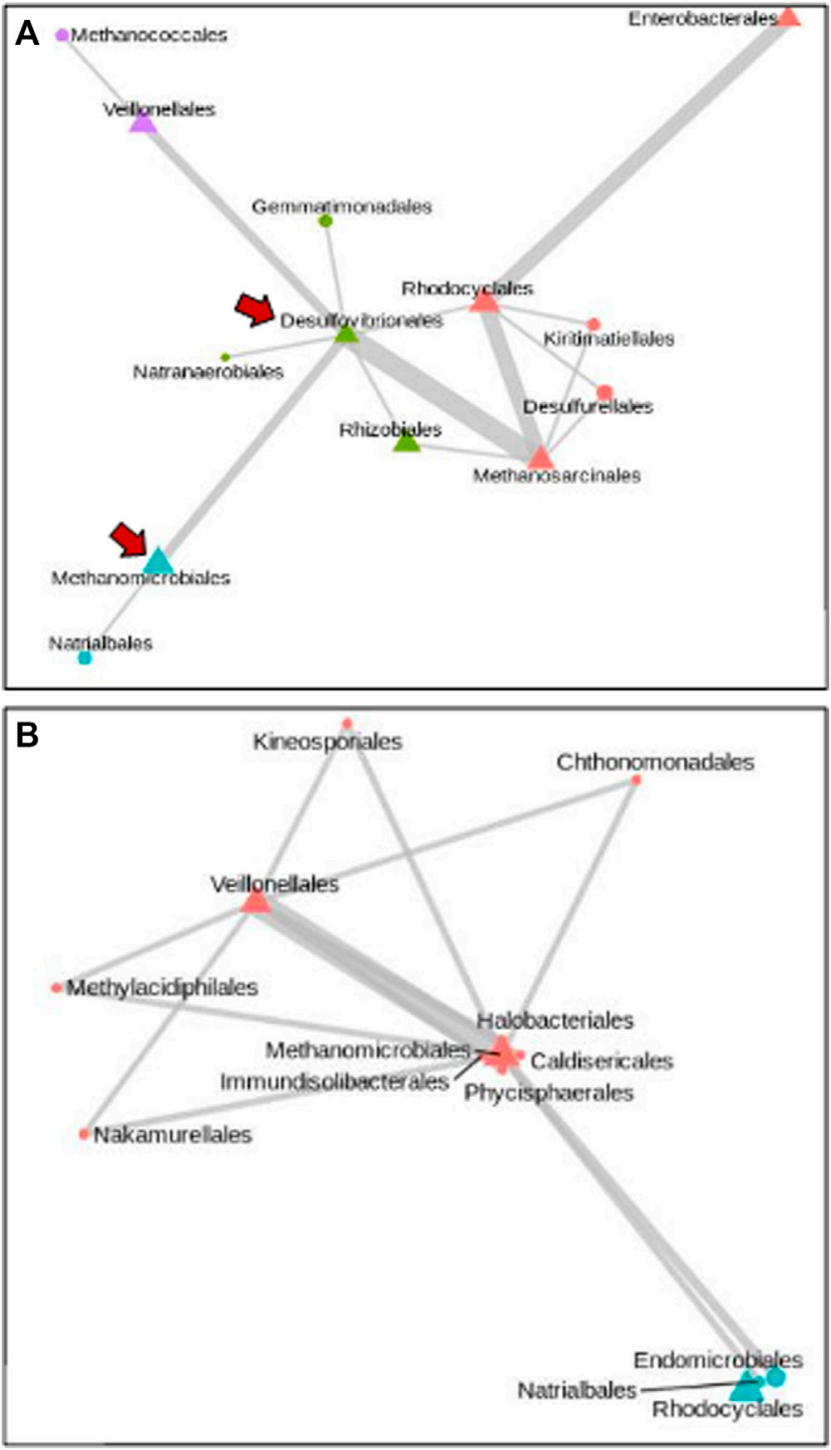
Network of taxa at Order level that are highly represented in high and low methane emitters **(A)** and **(B)**, respectively. The size of edges is proportional to the frequency of the association in ML networks. Node shape is indicative of high (triangle) or low (circle) abundance. In high methane emitters, H_2_-consuming bacterial orders are found highly abundant (**A**, arrows).

The ability to associate abundance levels of the most represented taxa from our Synomics Insights output to higher and lower trait values (here, respectively, cases and controls), allows us to perform comparative network analysis of bacterial taxa between the two groups. In these networks, the frequency at which a given taxon is found to be associated to another in the Synomics Insights networks can be represented by the thickness of connecting edges. The abundance of the taxon can be visualized with different node shapes for either highly abundant (bins 7–9) or very little abundant (bins 1–3) (Figure 3). Focusing on the highly represented orders of Desulfovibrionales and Veillonellales, which were found to be most representative among, respectively, high and low methane emitters, we found that Desulfovibrionales, an H_2_-consuming group of bacteria, were most often connected with methanogenic archea, such as Methanomicrobiales and Methanosarcinales (Figure 3A). Veillonella, which is known for lactate fermentation abilities in the gut of mammals, was found to be predominant in low-methane-emitter networks and associated with a number of other taxa including Methanomicrobiales, Halobacteriales, and others (Figure 3B).

#### Differentially Abundant Taxa

We used the original nonbinned table of relative abundance of order-level taxonomies to find differentially abundant taxa between high and low methane emitters. Using QIIME2 as a platform for analysis, we used two widely known tools for differential abundance analysis: linear discriminant analysis effect size (LEfSe) (Segata et al., 2011) and ANCOM (Mandal et al., 2015). With LEfSe using >2 as typical cutoff for the LDA score, we did not find any differentially abundant taxon between high and low emitters. ANCOM with default settings within QIIME2 only identified Methanosarcinales as highly abundant in low methane emitters (Supplementary Figure S7).

**FIGURE 4:**
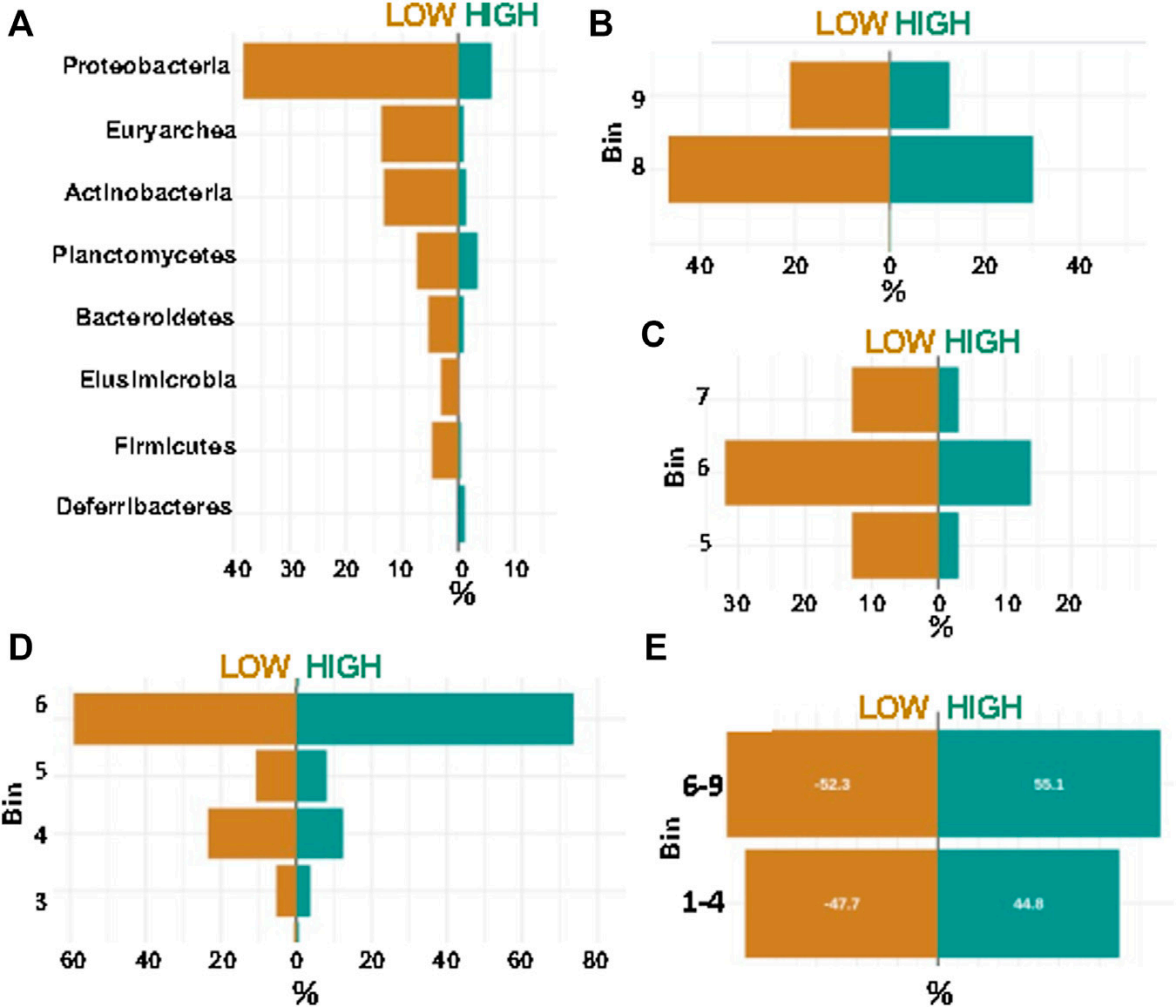
Percentage of top phyla called by Synomics Insights between low and high methane rumen samples, as overall proportion. **(A)** Phylum level analysis; **(B)** Order Veillonellales; **(C)** Order Methanobacteriales; **(D)** Order Desulfovibrionales; **(E)** Order Clostridiales. All differences between low and high emitters except for Clostridiales were significant at *p*-value < 10^–6^.

Using the networks of taxon:bin combinations from layer 4 (four combinations) of Synomics Insights, we grouped taxa in highly abundant (bins 7–9) or little abundant (bins 1–3) and statistically quantified differences in the abundance of these groups between low and high emitters either relatively to all the taxon:bin combinations within the same group or across all animals. We found much higher abundance of the phylum Proteobacteria in low vs. high methane samples in our results (Figure 4A, *p*-value < 10^–6^) and in high milk producers (Supplementary Figure S8). Order-level analysis found higher abundance of Veillonellales and Methanobacteriales among low emitters (Figures 4B,C, *p*-value < 10^–6^). Veillonella and proteobacteria have been previously found to be associated with the rumen of low-methane-emitting cattle (Wallace et al., 2015). Shifting focus on high methane emitters, the output from Synomics Insights presented a high proportion of above-average abundance (bin >5) of Desulfovibrionales compared with low emitters (Figure 4D, *p*-value < 10^–6^). Also, Clostridiales were more abundant in high methane emitters compared with low emitters (Figure 4E). Overall, 21/26 orders shared between low and high methane emitters were found significantly differentially abundant. At the species level, Synomics Insights showed higher abundance of *Megasphaera elsdenii* in low compared with high methane samples (Supplementary Figure S9). Importantly, other studies show that rumen samples from low-emitting cows typically show higher lactate and succinate-producing taxa (Kittelmann et al., 2014). *Megasphaera* is an ecologically important rumen bacterium that metabolizes lactate, relieving rumen acidosis induced by a high-grain diet (Chen et al., 2019).

**FIGURE 5:**
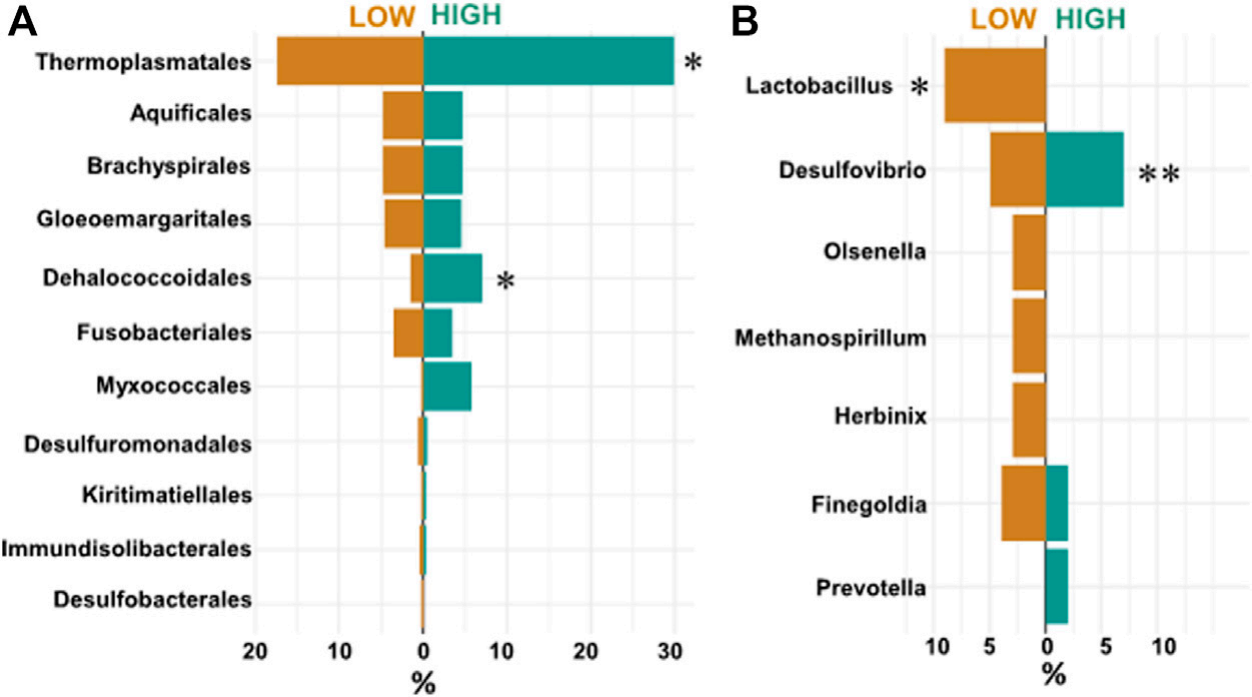
**(A)** Proportions of top enriched (bins 7–9) orders called by Synomics Insights in low and high RFI samples. Thermoplasmatales (*p*-value > 0.1), Dehalococcales (*p*-value < 0.1) seem enriched specifically in high RFI (*). **(B)** Counts of enriched (bins 6–9) genuses identified by Synomics Insights in layer two using species-level input. While *Lactobacillus* seems associated uniquely with low RFI samples (*, *p*-value < 0.05), Desulfovibrio, a marker for high methane rumen, was found associated to higher RFI samples (**, *p*-value = 0.578).

Shifting focus on RFI as a trait, our Synomics Insights platform found that high RFI samples were characterized by a higher abundance of taxa linked to high H_2_ levels, which is a typical marker for high methane production (Wang et al., 2014). Specifically, 11 taxa were identified in the output; however, among taxa characterized by more abundant levels (bins 7–9), only Thermoplasmatales and Dehalococcoidales were found enriched in high RFI samples (Figure 5A) although the former not at a significant *p*-value threshold. At lower taxonomic levels, a higher abundance of the genus Desulfovibrio was found associated with high RFI output (Figure 5B), whereas Lactobacillaceae, such as *Lactobacillus*, were found significantly associated with low RFI output (Figure 5B, *p*-value < .1) (Murad, 2019; Bergamaschi et al., 2020).

#### GWAS of Heritable Core Microbiome

**FIGURE 6:**
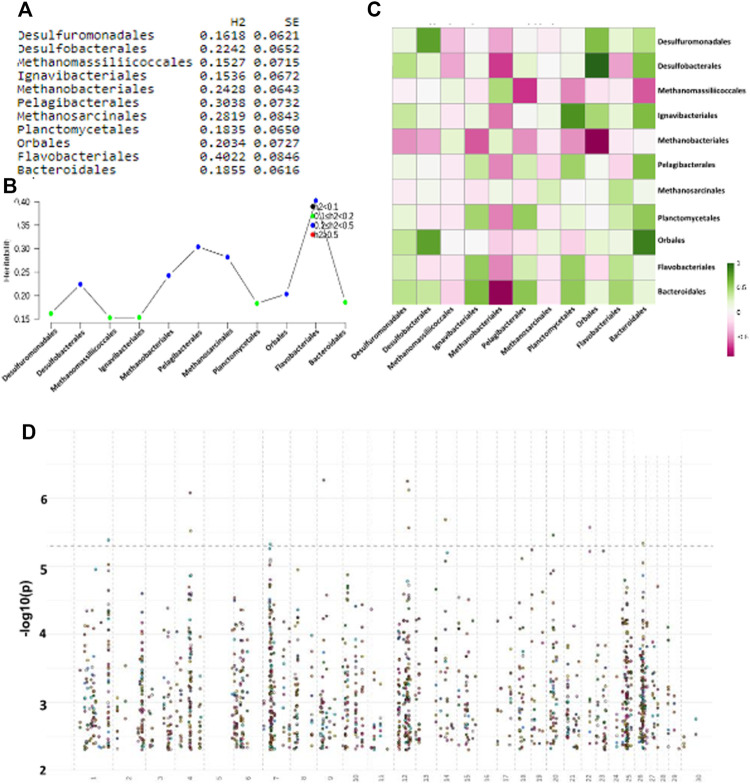
**(A)**: List of heritable bacterial orders with St. Error (SE); **(B)** Plot showing strength of heritability across taxonomies; **(C)** Phenotypic and genetic correlations (*r* ^2^) of heritable orders (respectively lower-left and upper-right half-quadrant); **(D)** Manhattan plot of GWAS using each of the 11 taxonomic orders individually as trait.

Heritability was measured on all those taxa from the original set of 150 order-level taxonomies that were found in at least 70% of animals. Only a core 73 taxa passed this strict requirement, and of these, 11 were found to be heritable (*h* ^*2*^ > 0.15) (Figures 6A,B). An analysis of the phenotypic and genetic correlation of animal hosts using these 11 taxonomies shows some important clues on the type of biological relationship among these taxonomic orders. Methanobacteriales are generally negatively correlated with a number of other bacterial orders and especially Bacteroidales and Orbales, whereas Desulfobacterales are strongly positively correlated with Orbales and Desulfuromonadales (Figure 6C). Similarly to Methanobacteriales, which were positively associated, Methanomassiliicoccales, a taxonomic order of archaea that enclose several methanogenic organisms found in the animal and human intestinal tract, were negatively correlated with Bacteroidales (Figure 6C). Relative abundance levels of Methanobacteriales and Methanomassiliicoccales are reported to change significantly in rumen samples from animals with different methane emission and feed efficiency values (Bowen et al., 2020). Our results suggest a potential competition within the rumen bacterial community between methanogenic archea and gamma and deltaproteobacteria.

GWAS analysis was performed using PLINK1.9 with default parameters (See Materials and Methods section). We found 307 SNPs with a *p*-value <10^–2^, whereas no SNP passed a more stringent cutoff for GWAS analysis (<10^–8^) and were relatively distributed across all chromosomes with some host spots on chromosomes 7, 12, 25, and 26 (Figure 6D). We focused on five of the core taxonomic orders that strongly correlated among themselves and that have previously demonstrated links to methane metabolism: Desulfurobacterales, Methanomassiliicoccales, Methanobacteriales, Orbales, and Bacteroidales. We found 44 GWAS SNPs that were shared among these five orders, and 22 of these were within protein-coding genes. A functional enrichment analysis using DAVID (Huang et al., 2009) found the “intracellular” GO term with significant Benjamini-corrected *p*-value (*p* = .23), which included Annexin A5, MYZAP (Adherin), poly-(ADP-ribose)-polymerase and a potassium channel (Supplementary Figure S10).

#### Synomics Insights Host Genetic Determinants of Microbiome Composition

**FIGURE 7:**
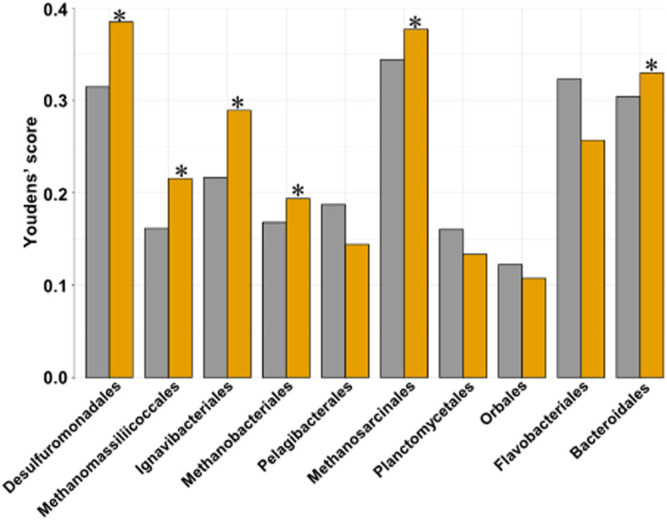
Bar plot representing the Youden’s J statistic (Materials and Methods) of the predictive ability of the best 100 SNP:genotypes from respectively GWAS (grey) and our Synomics Insights (orange). In 6/10 of the tested taxa our core engine performs better compared to GWAS (*).

To find host SNP:genotype networks linked to changes in the core 11 heritable bacterial orders through Synomics Insights, we normalized and binarized each taxon as for phenotype traits, obtaining a set of cases and controls in which cases had high abundance and controls low abundance for the given taxonomy. Synomics Insights finds networks of SNP:genotype combinations that are significantly enriched in cases vs. controls. We compared the predictive ability of the best SNP:genotype combinations obtained from our platform to the best obtained from GWAS and found that in 6/10 of the taxa tested (Desulfurobacterales had too little SNPs for comparison), the SNP:genotype combinations identified by Synomics Insights were better classifiers of cases and controls compared with GWAS SNPs (Figure 7). This result suggests a stronger biological relevance of Synomics Insights’ genetic features compared with those obtained with GWAS.

There was not a great amount of overlap 4) between GWAS SNPs and those identified by Synomics Insights using the 11 core heritable microbial orders as trait. A total of 1290 SNP:genotype combinations were identified exclusively by our platform. Among these SNPs, 376 were shared by nine of the 11 heritable orders of bacteria, including Methanomassiliicoccales, and 113 were shared by >90% of all the 73 core taxonomies, including those not very heritable. Of the 376 SNPs present in the core heritable bacteria, 117 mapped in protein coding genes. A functional enrichment analysis (DAVID) performed with these genes did not show significant pathways or functions; however, it did present a significant enrichment in nucleotide binding properties (Supplementary Figure S11A). Using all protein-encoding genes in which the original 1,293 SNPs were found, we observed a significant enrichment of coiled-coil-containing proteins involved in cell division and cell cycle. We also found a significant enrichment (Benjamini corrected *p*-value = .58) for proteins containing a TBC (Tre-2/Bub2/Cdc12) domain (Supplementary Figure S11B).

## Discussion

Proving a direct relationship between changes in heritable members of the rumen microbial population and methane production or nutrient utilization has not been easy. It is well established that complex traits are normally caused by many genetic determinants often with very little individual effects. Furthermore, the influence of host genetics is entwined with confounding effects such as diet, geographical location, and others (Henderson et al., 2015). Whereas host additive genetic effects undoubtedly influence rumen bacterial and archaeal taxa, these effects are often too weak to be meaningful (Difford et al., 2018). However, recent research shows that immunity-associated bacteria are closely linked to host SNPs located in genes involved in energy metabolism and immunity, further providing evidence of the modulation of microbiota structure by host genetics (Fan et al., 2021). In this study, authors further suggest that the gut microbiota structure could be influenced by host genetics throughout life.

Several ML techniques have been applied mostly to human microbiome data, the most common being random forest, support vector machines, logistic regression, and k-NN. However, underrepresented ML tools, such as DL, spatiotemporal, and dynamic modeling, methods for longitudinal and mechanistic analyses or integrative methods for data from different sources could prove key to understand microbiome–host and microbiome–disease interactions. Here, we set out to investigate whether our in-house-developed ML platform Synomics Insights was better at identifying cues that could demonstrate the action of specific sets of host SNPs or genes on rumen bacteria responsible for methane production and RFI in cattle. Therefore, our assumption at the onset of the study is that changes in the abundance of certain bacteria drive differences in methane emission and RFI, and these changes are modulated by a finite and detectable number of host SNPs and genes.

Synomics Insights was utilized to analyze both microbiome and host genotype data. For microbiome analysis, we fed a large number of microbial taxa, some of which showed significant h^2^ values, and their abundance levels, into the core combinatorial analysis engine of Synomics Insights. We showed that Synomics Insights can identify taxonomy-abundance combinations in microbiome data that can classify case/control sample groups significantly better than other publicly available tools. Additionally, this combinatorial methodology can identify significant differences in the abundance of relevant microbial taxa that are difficult to detect. For methane emission, the study identified 26 order-level taxa shared by high and low emitters that are part of networks our core engine reported as significantly enriched. A Z-test on proportions found 21/26 (81%) of these taxa were differentially enriched between high and low methane emitters (*p*-value < 0.05). In particular, the phylum of Proteobacteria and the order Desulfovibrionales were found enriched in high emitters, and the order Veillonellales was found more abundant in low emitters as previously reported for cattle (Wallace et al., 2015). Importantly, only the order Methanosarcinales could be identified as differentially abundant using the publicly available tool ANCOM (Mandal et al., 2015). The analysis of RFI proved more complex as the signal (networks of SNP:genotype combinations) obtained by running Synomics Insights was noisy based on its ability to predict cases/controls. Nevertheless, downstream analysis showed several order-, genus-, and species-level taxa enriched in networks. At the order level, 11 taxa were identified, but only Dehalococcales (*p*-value < .1) was significantly enriched among high RFI samples. At the genus level, several taxa were found in networks albeit with relatively low counts. Among these, the most abundant *Lactobacillus* was enriched among low RFI samples (*p*-value < .1). Lactobacilli are found associated with food efficiency in swine (Bergamaschi et al., 2020), and their use in probiotics is found to improve feed efficiency and productivity in dairy cattle (Murad, 2019).

The second phase of our analysis dealt with finding host SNPs significantly associated with changes in heritable rumen bacteria, some of which with clear links to methanogenesis. There are no reports in literature of what genetic factors in ruminants modulate the rumen bacteria responsible for methanogenesis, and most of the studies available only quantify the amount of variation in methane emission or microbiota structure explained by host genetics. We compared Synomics Insights technology with GWAS, the mainstream methodology for association studies. Here, we show that, through the testing of millions of SNP:genotype combinations, Synomics Insights identifies predictors of rumen bacterial abundance as both individual SNPs and networks of SNPs that would otherwise be discarded as poor predictors when considered individually, for example, in a GWAS approach. Indeed, 1,290 additional SNPs that were not found by GWAS were significantly associated to changes in heritable bacterial taxa by Synomics Insights. We did not observe SNPs located in QTLs previously linked to feed efficiency traits (de Oliveira et al., 2014; Li et al., 2019) or in genes with a role in the host immune responses known to interact with the gut microbial flora (Tomkovich and Jobin, 2016). However, we found significant enrichment for molecular signatures linked to gene expression regulation and an enrichment for biological functions connected to cell division and homeostasis.

## Conclusion

The advantages of ML and feature network-based methods over classical statistical-bioinformatics methods in the handling and analysis of multidimensional data are numerous, especially with regards to the inference of relationships between variables for automatic pattern discovery and biomarker detection. These methods have only recently started to be applied to microbiome data with very promising results (Marcos-Zambrano et al., 2021). In this study, we show that, by using these approaches that are part of Synomics Insights platform, we could obtain a deeper representation and quantification of changes in the bacterial composition of rumen microbiome samples across methane emission and feed efficiency traits for sustainable and resource efficient animal production. Compared with using conventional statistical methods, this strategy led to the identification of specific groups of bacteria that can be used as targets or biomarkers for methane emission in dairy cattle. We could not obtain such novel and biologically relevant targets or biomarkers using other standard and tested methodologies when applied to the data set we used here.

By harnessing its advanced omics feature discovery method and its computational power designed to test hundreds of millions of potential high order interactions among features, for example, the Synomics Insights platform could help unlock the host’s genetic as well as microbiome potential in precision management and genetic selection programs. This study shows specific application of this technology to make beneficial changes in complex traits, such as methane production by rumen microbes.

## Data Availability

Publicly available datasets were analyzed in this study. This data can be found here: https://www.science.org/doi/10.1126/sciadv.aav8391 and https://www.ncbi.nlm.nih.gov/bioproject/?term = prjna517480.
