# Supplementary material for: Host Genome–Metagenome Analyses Using Combinatorial Network Methods Reveal Key Metagenomic and Host Genetic Features for Methane Emission and Feed Efficiency in Cattle
Source: Front Genet. 2022 Feb 23;13:795717. doi: 10.3389/fgene.2022.795717 (PMC8905538; doi:10.3389/fgene.2022.795717)
Supplement: Supplementary file 1 [file DataSheet1.PDF]

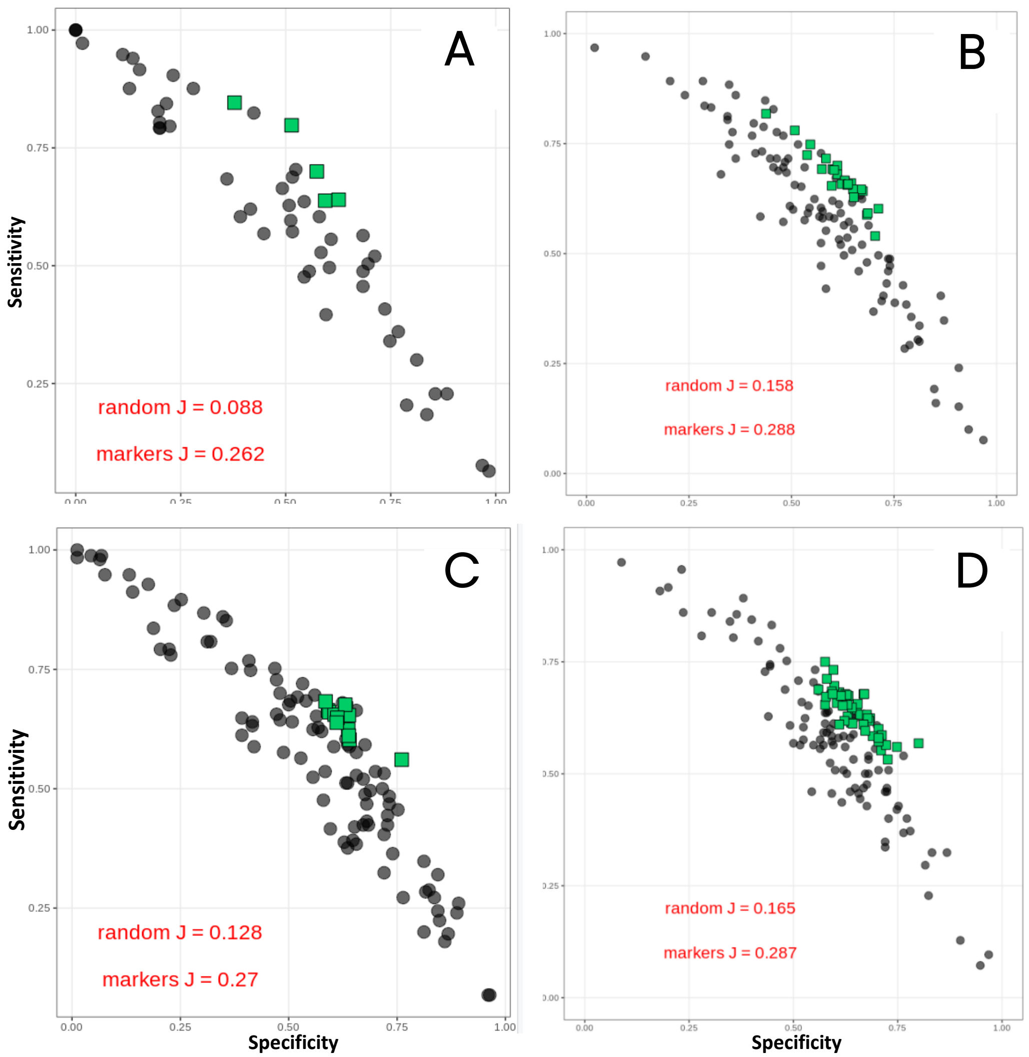

Sup. Fig. 1 Sensitivity/specificity plots of predictions of cases and controls for methane emission using taxa at Order taxonomic level. Cases and controls were defined as top and bottom 25% percentile. Green squares: taxa obtained from Synomics Insights, Grey circles: random bootstrap selection (A-C: layer 1 to 4).

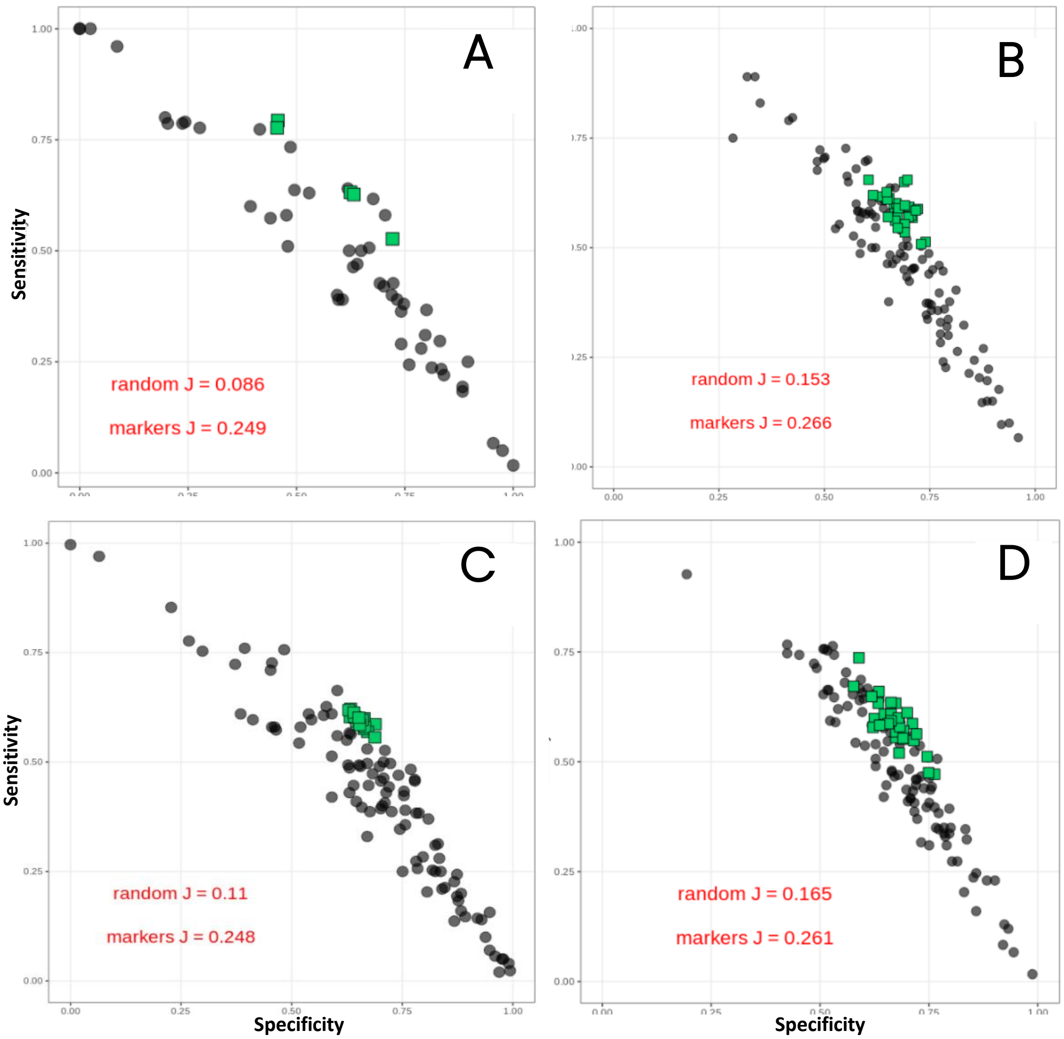

Sup. Fig. 2 Sensitivity/specificity plots of predictions of cases and controls for methane emission using taxa at Order taxonomic level. Cases and controls were defined as Z-scores ± 0.5 from the mean. Green squares: taxa obtained from Synomics Insights, Grey circles: random bootstrap selection (A-C: layer 1 to 4).

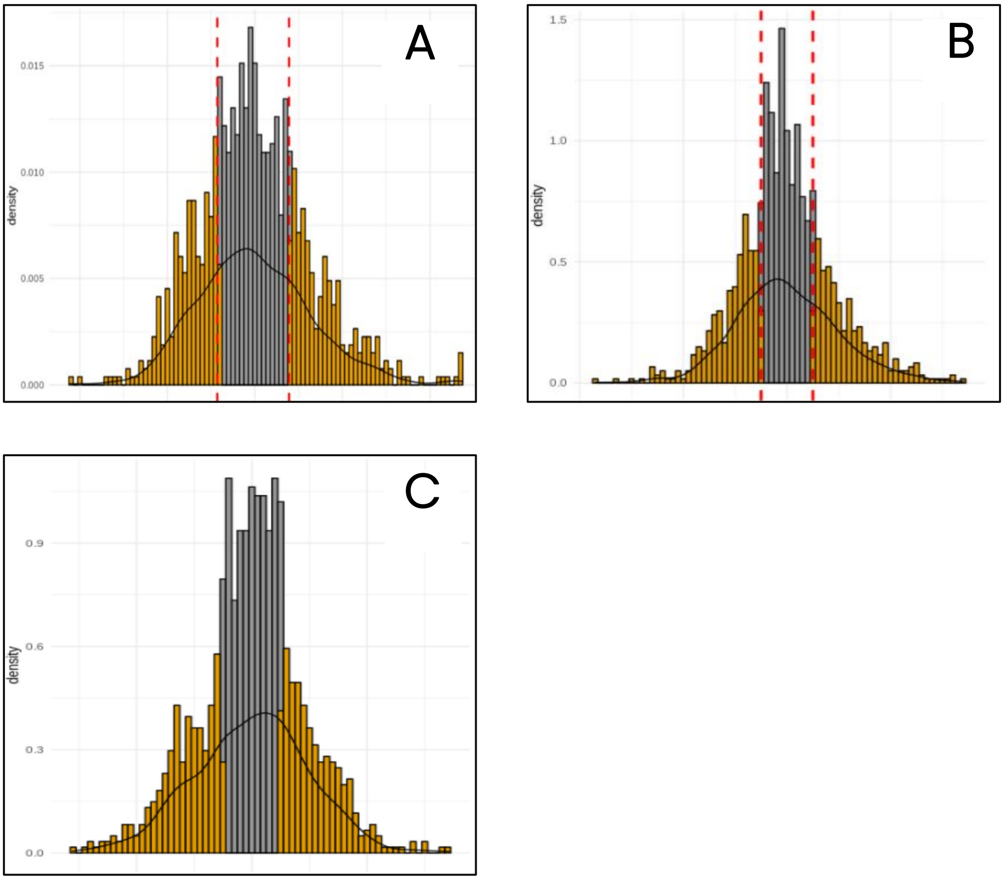

Sup. Fig. 3 Z-score normalization and segmentation of datasets in cases (right tail) and controls (left tail) for methane (A, 623 samples) milk yield (B, 604 samples) and RFI (C, 606 animals).

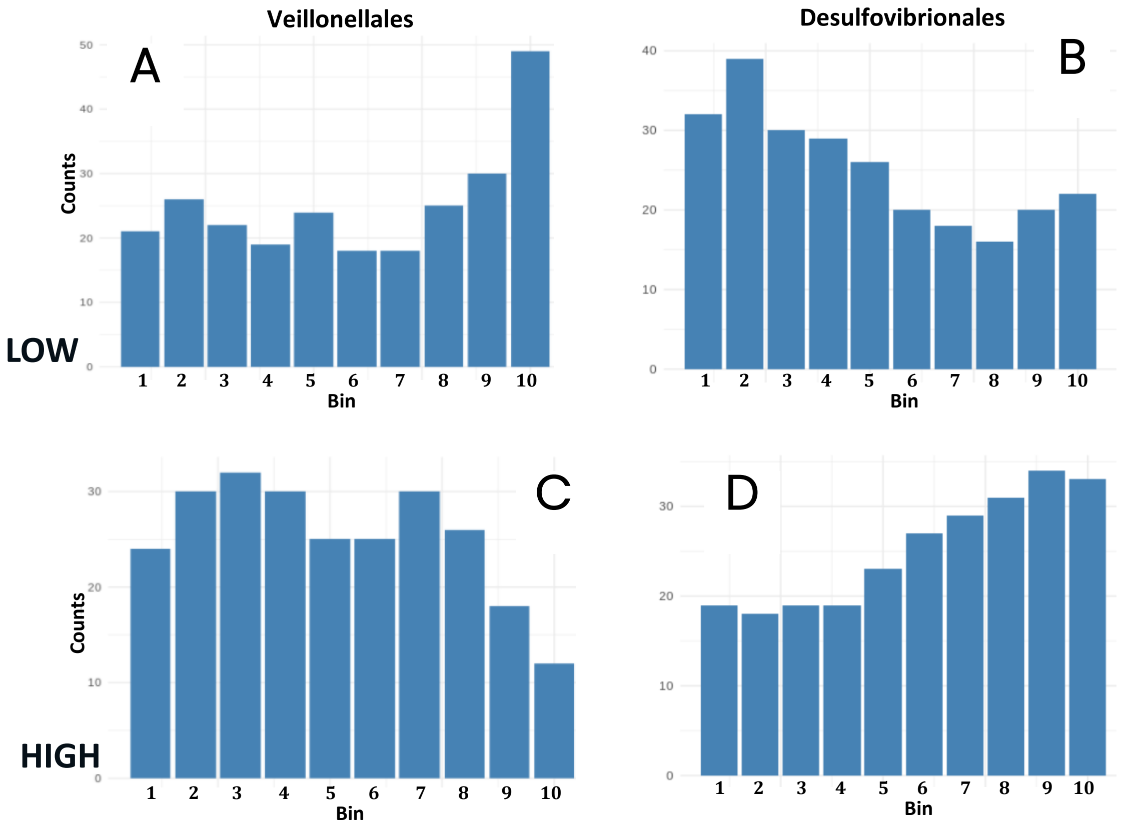

Sup. Fig. 4 Density distribution of bins in the dataset for two key bacterial orders: Veillonellales (A, C) and Desulfovibrionales (B, D) for low methane emission (A, B) and high methane emission (C, D).

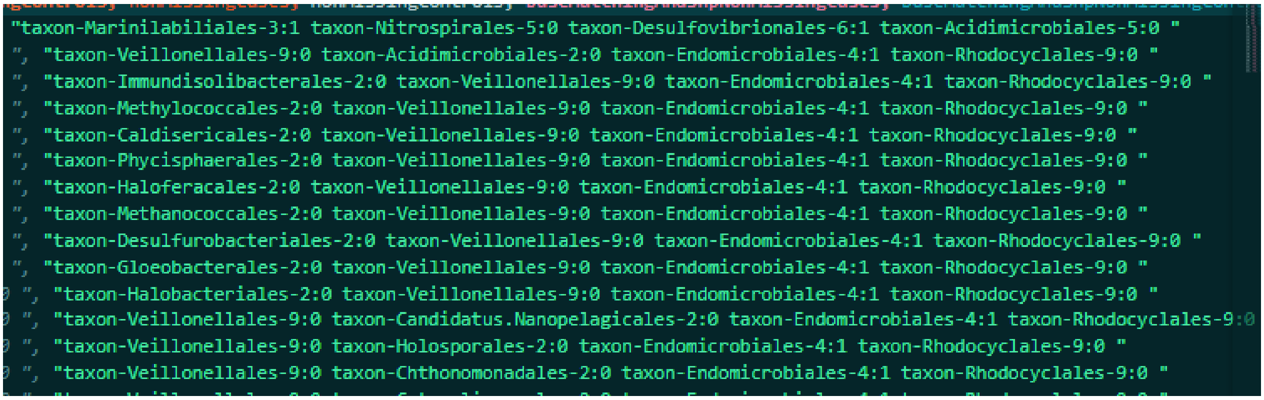

"taxon-Marinilabiliales-3:1 taxon-Nitrospirales-5:0 taxon-Desulfovibrionales-6:1 taxon-Acidimicrobiales-5:0 "
", "taxon-Veillonellales-9:0 taxon-Acidimicrobiales-2:0 taxon-Endomicrobiales-4:1 taxon-Rhodocyclales-9:0 "
", "taxon-Immundisolibacterales-2:0 taxon-Veillonellales-9:0 taxon-Endomicrobiales-4:1 taxon-Rhodocyclales-9:0 "
", "taxon-Methylococcales-2:0 taxon-Veillonellales-9:0 taxon-Endomicrobiales-4:1 taxon-Rhodocyclales-9:0 "
", "taxon-Caldisericales-2:0 taxon-Veillonellales-9:0 taxon-Endomicrobiales-4:1 taxon-Rhodocyclales-9:0 "
", "taxon-Phycisphaerales-2:0 taxon-Veillonellales-9:0 taxon-Endomicrobiales-4:1 taxon-Rhodocyclales-9:0 "
", "taxon-Haloferacales-2:0 taxon-Veillonellales-9:0 taxon-Endomicrobiales-4:1 taxon-Rhodocyclales-9:0 "
", "taxon-Methanococcales-2:0 taxon-Veillonellales-9:0 taxon-Endomicrobiales-4:1 taxon-Rhodocyclales-9:0 "
", "taxon-Desulfurobacteriales-2:0 taxon-Veillonellales-9:0 taxon-Endomicrobiales-4:1 taxon-Rhodocyclales-9:0 "
", "taxon-Gloeobacterales-2:0 taxon-Veillonellales-9:0 taxon-Endomicrobiales-4:1 taxon-Rhodocyclales-9:0 "
", "taxon-Halobacteriales-2:0 taxon-Veillonellales-9:0 taxon-Endomicrobiales-4:1 taxon-Rhodocyclales-9:0 "
", "taxon-Veillonellales-9:0 taxon-Candidatus.Nanopelagicales-2:0 taxon-Endomicrobiales-4:1 taxon-Rhodocyclales-9:0
", "taxon-Veillonellales-9:0 taxon-Holosporales-2:0 taxon-Endomicrobiales-4:1 taxon-Rhodocyclales-9:0 "
", "taxon-Veillonellales-9:0 taxon-Chthonomonadales-2:0 taxon-Endomicrobiales-4:1 taxon-Rhodocyclales-9:0 "

Sup. Fig. 5 Snapshot of layer 4 taxon:bin networks from Synomics Insights.

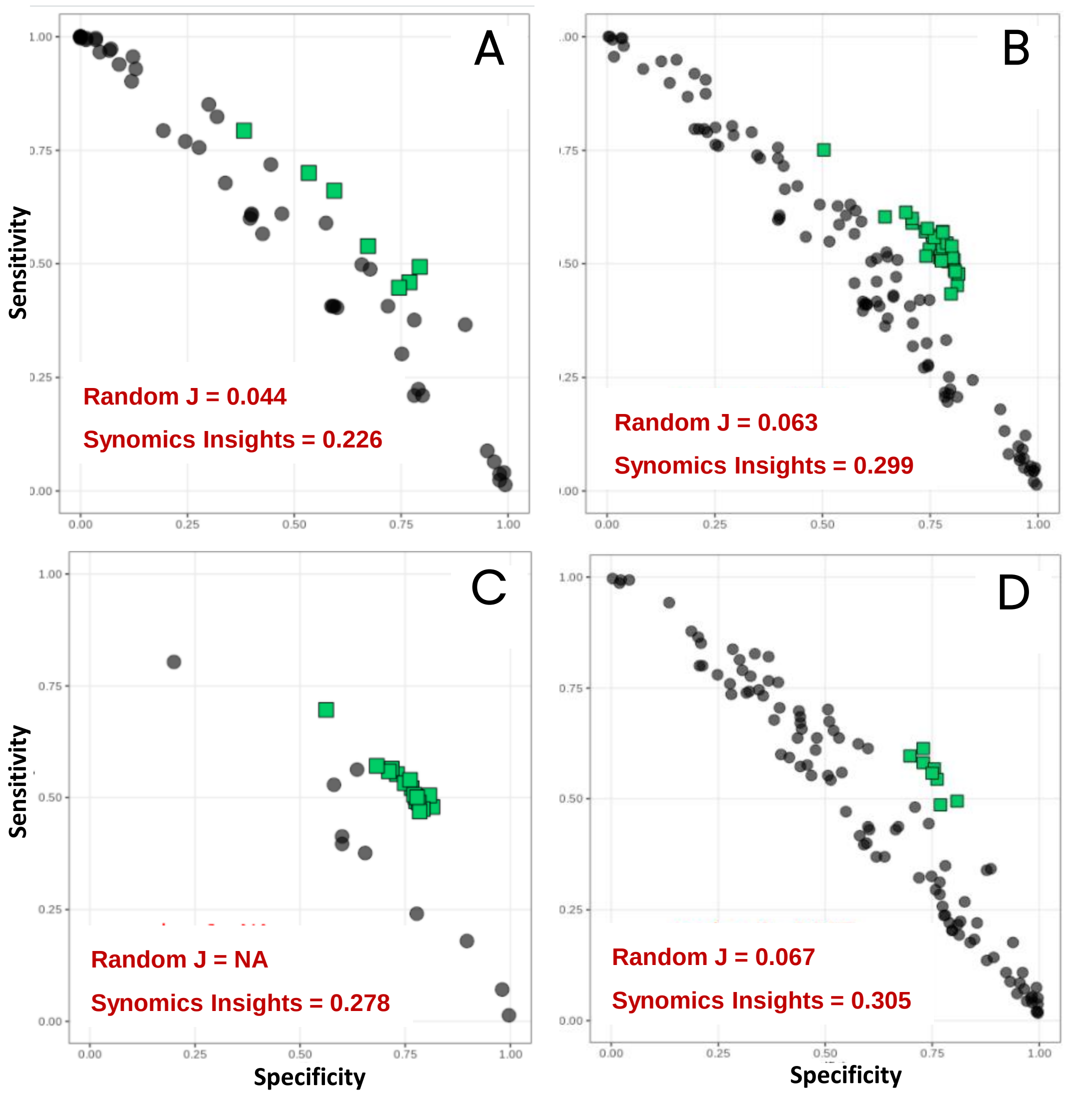

Sup. Fig. 6 Top taxon:bin combinations at Species level for milk yield from layers 1-4 (A-C, green). Synomics Insights output shows markedly higher predictive accuracy compared to random (grey) as measured with a Youden's statstic (J) (Materials and Methods).

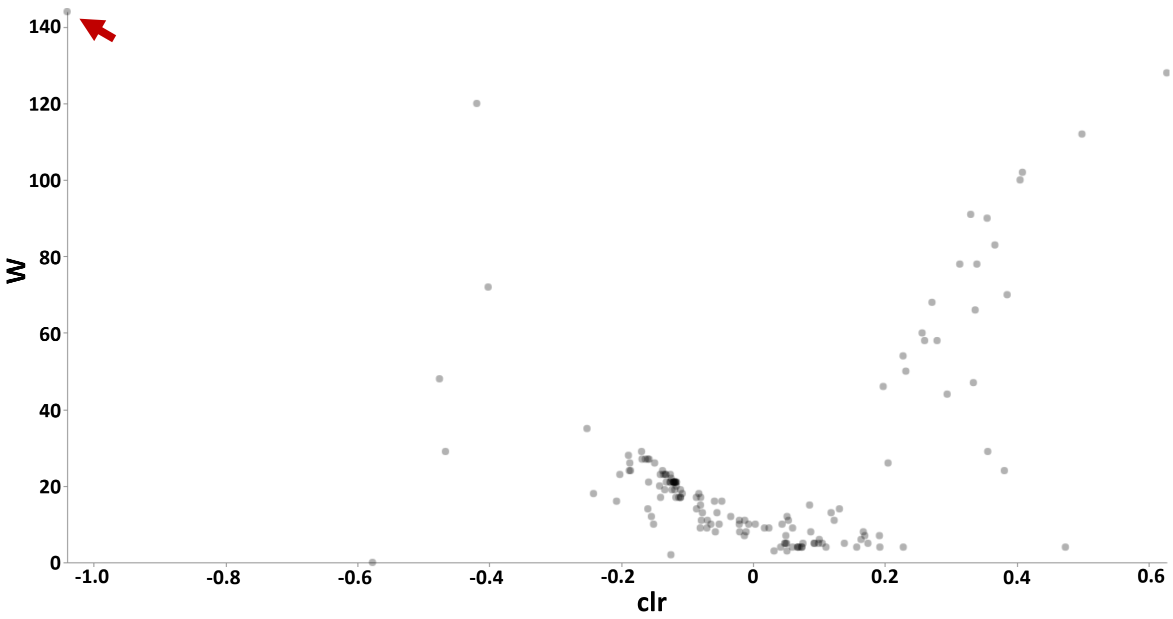

Sup. Fig. 7 ANCOM analysis of differentially abundant taxa between low and high methane emitters. Only Methanosarcinales is found significantly more abundant in low methane emitters (arrow) (W: number of rejected pairwise tests or null hypotheses; clr: transformed mean difference between cases and controls. A positive value represents higher abundance in cases, a negative a higher abundance in controls).

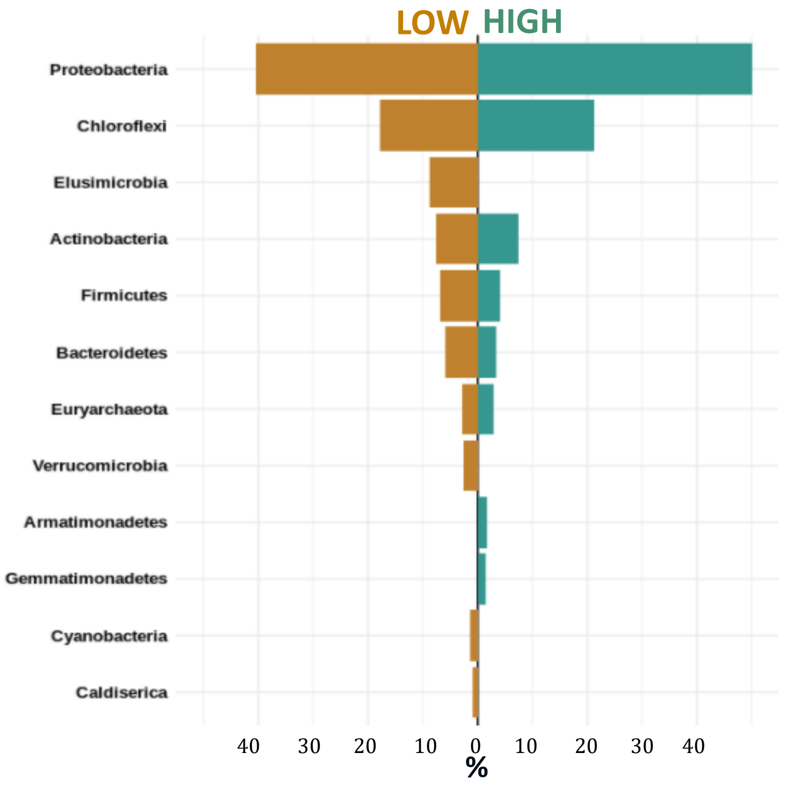

Sup. Fig. 8. Relative proportion of Phyla called by Synomics Insights in low (brown) vs high (cyan) milk yield.

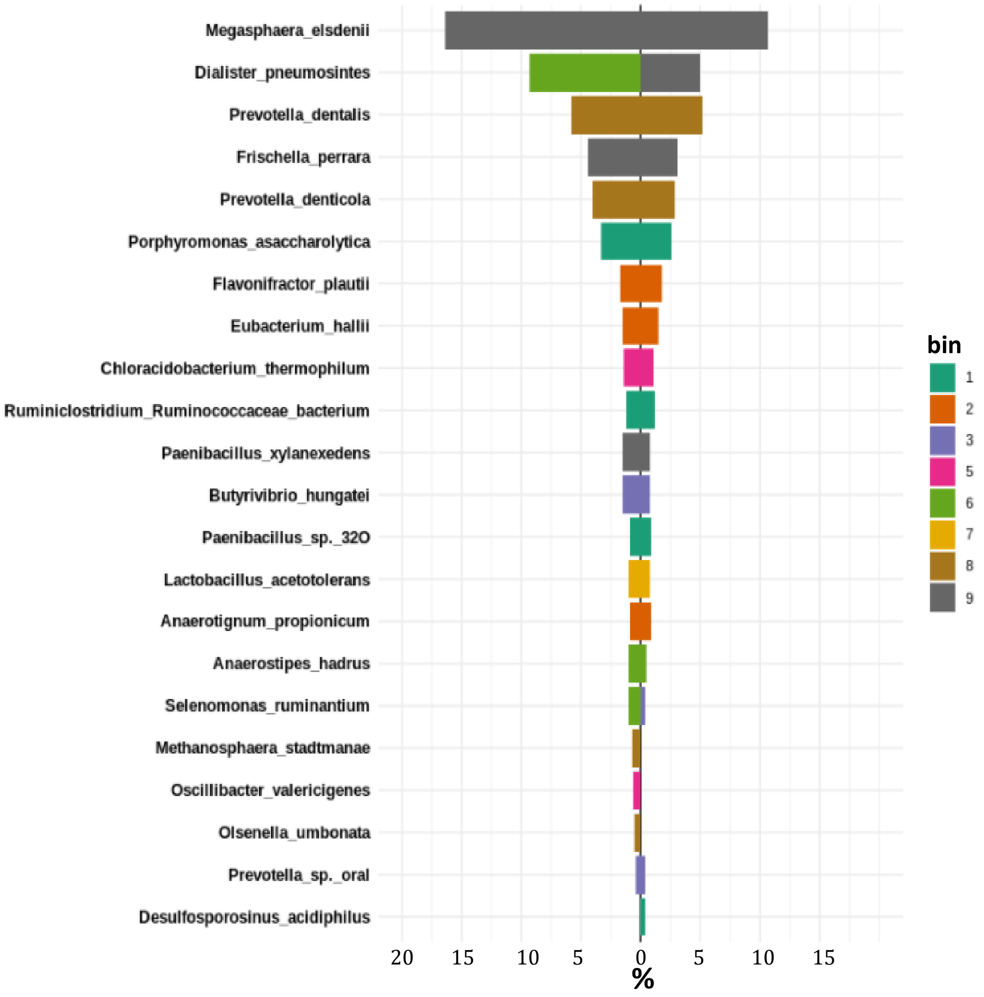

Sup. Fig. 9. Relative proportion of species called by Synomics Insights in low vs high methane samples, and their abundance as specified by colour (bin: 1 = lowest abundance, 9 = higher abundance).

Category	Term	RT	Genes	Count	%	P-Value	Benjamini
GOTERM_CC_DIRECT	intracellular	RT		4	28.6	9.3E-3	2.3E-1
GOTERM_BP_DIRECT	potassium ion transmembrane transport	RT		2	14.3	3.9E-2	1.0E0
GOTERM_CC_DIRECT	voltage-gated potassium channel complex	RT		2	14.3	4.5E-2	5.7E-1

4 record(s)

ID	GENE NAME
annexin A5(ANXA5)	annexin A5(ANXA5)
myocardial zonula adherens protein(MYZAP)	myocardial zonula adherens protein(MYZAP)
poly(ADP-ribose) polymerase family member 8(PARP8)	poly(ADP-ribose) polymerase family member 8(PARP8)
potassium voltage-gated channel subfamily B member 2(KCNB2)	potassium voltage-gated channel subfamily B member 2(KCNB2)

Sup. Fig. 10. Functional cluster with Pvalue for GWAS SNPs mapping to protein coding genes. Lower panel: genes included in the 'intracellular' term.

A

ENSEMBL_GENE_ID	GENE NAME
ENSBTAG00000002747	ATP binding cassette subfamily A member 5(ABCA5)
ENSBTAG00000038523	ATPase Na+/K+ transporting subunit alpha 4(ATP1A4)
ENSBTAG00000007093	DEAD/H-box helicase 11(DDX11)
ENSBTAG00000027795	G elongation factor mitochondrial 1(GFM1)
ENSBTAG00000002174	RAS like estrogen regulated growth inhibitor(RERG)
ENSBTAG00000008963	citron rho-interacting serine/threonine kinase(CIT)
ENSBTAG00000011820	death associated protein kinase 2(DAPK2)
ENSBTAG00000002744	muscle associated receptor tyrosine kinase(MUSK)
ENSBTAG00000021538	myosin IE(MYO1E)
ENSBTAG00000002123	myosin IIIA(MYO3A)
ENSBTAG00000026290	phosphatidylinositol 3-kinase catalytic subunit type 3(PIK3C3)
ENSBTAG00000003276	protein kinase C eta(PRKCH)
ENSBTAG00000010906	ribosomal protein S6 kinase A5(RPS6KA5)
ENSBTAG00000018785	tubulin beta 1 class VI(TUBB1)

B

ENSEMBL_GENE_ID	GENE NAME
ENSBTAG00000013699	TBC1 domain family member 1(TBC1D1)
ENSBTAG00000005493	TBC1 domain family member 14(TBC1D14)
ENSBTAG00000033214	TBC1 domain family member 19(TBC1D19)
ENSBTAG00000015611	TBC1 domain family member 9B(TBC1D9B)

Sup. Fig. 11. **A)** Results of DAVID (Huang et al., 2009) functional enrichment for the protein-coding genes containing the 117 SNPs found in core heritable bacterial orders; **B)** Functional enrichment utilizing the protein coding genes containing all 1293 SNPs from Synomics Insights.
